# First genome sequence of a European *Alternaria brassicae* isolate and genes involved in early development of alternaria leaf spot on *Brassica juncea*

**DOI:** 10.1002/ps.70477

**Published:** 2026-01-21

**Authors:** Kevin M. King, Graham McLaughlin, Chinthani S. Karandeni Dewage, Zedi Gao, David J. Hughes, Jonathan S. West, Henrik U. Stotz

**Affiliations:** ^1^ Rothamsted Research West Common Hertfordshire UK; ^2^ School of Health, Medicine and Life Sciences University of Hertfordshire Hertfordshire UK

**Keywords:** comparative genomics, dual RNA‐sequencing, host–pathogen interactions, OPPP, pathogenesis‐related proteins, photosystem suppression

## Abstract

**BACKGROUND:**

*Alternaria brassicae* is a necrotrophic fungal pathogen causing grey leaf spot disease in Brassicaceae crops, notably *Brassica juncea*. While previous genomic studies have focused on Indian isolates, the molecular basis of host–pathogen interactions in European isolates remains unexplored.

**RESULTS:**

We report the first genome sequence of a UK *A. brassicae* isolate (AA1/5), assembled using PacBio and Illumina platforms, revealing a 32.6 Mb genome with 7228 predicted genes. Comparative analysis with the Indian J3 isolate suggests genomic divergence, including fewer repetitive elements and secreted proteins in AA1/5. Dual RNA‐sequencing profiling of AA1/5 and two *B. juncea* cultivars (Sej‐2 (2) and Pusa Jaikisan) identified differential expression of fungal genes involved in carbohydrate metabolism, cell wall degradation, and endocytosis. Host transcriptomics revealed suppression of photosystem genes and induction of oxidative pentose phosphate pathway genes. Cultivar‐specific responses included differential regulation of defence‐related genes such as *PR‐3*, *RLP35*, *JOX4*, and *CYP81F3*. Quantitative reverse transcriptase‐PCR validated the host transcriptomic dataset with up‐regulation after fungal infection in both cultivars of two pathogenesis‐related genes, *PR‐4* and *PR‐5*.

**CONCLUSION:**

This study provides novel insights into the genomic and transcriptomic landscape of a European *A. brassicae* isolate and its interaction with *B. juncea*. The findings highlight conserved and divergent pathogenicity mechanisms and host responses, offering a foundation for future resistance breeding and functional studies in Brassicaceae crops. © 2026 The Author(s). *Pest Management Science* published by John Wiley & Sons Ltd on behalf of Society of Chemical Industry.

## Graphical abstract

This article reports the first genome sequence of a UK *Alternaria brassicae* isolate. Dual RNA‐sequencing profiling of *A. brassicae*‐infected *Brassica juncea* leaves identified differentially expressed genes involved in pathogenicity and host response pathways in moderately resistant Sej‐2 (2) and moderately susceptible Pusa Jaikisan cultivars.

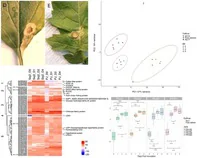

## 1 INTRODUCTION

*Alternaria brassicae* (Berk.) Sacc. is a necrotrophic fungal pathogen with a host range limited to the Brassicaceae. Toxins are thought of as being responsible for delimiting the host range of *Alternaria* spp.^1^ In the case of *A. brassicae*, several toxins, cyclic depsipeptides phytotoxins like destruxin B and the protein ABR‐toxin, are produced.^2^, ^3^ However, in contrast to destruxin B, the ABR‐toxin operates as a truly host‐selective toxin; ABR‐toxin is produced in spore germination fluids only on the surface of host plants and facilitates initial pathogen colonisation.^3^, ^4^, ^5^ *A. brassicae* causes grey leaf spot disease and severe yield losses on mustard rape (*Brassica juncea*) in India, and on oilseed rape (*Brassica napus*) in different parts of the world.^6^

Various wild *Brassica* relatives have been identified with sources of resistance against *A. brassicae* for introgression into cultivated *Brassica* crops.^7^ Resistance of *Camelina sativa* and *Sinapis alba* against *A. brassicae* is believed to involve rapid induction and accumulation of phytoalexins after pathogen challenge.^8^, ^9^ Transgenic approaches have been used to increase resistance against *A. brassicae*. Constitutive expression of hevein, a chitin‐binding lectin from the rubber plant *Hevea brasiliensis*, was shown to protect *B. juncea* against *A. brassicae*.^10^ Similarly, heterologous pathogenesis‐related (PR) genes have been used to increase resistance of *B. juncea* against *A. brassicae*.^11^, ^12^

Genome‐wide analyses have included sequencing of the *A. brassicae* genome and transcriptomics of the pathogen during infection of susceptible *B. juncea* cv. Varuna.^13^ The genome of an *A. brassicae* isolate collected from a field in Delhi, India was sequenced using Oxford Nanopore technology.^14^ The total size of the genome was 34.1 Mb with 11 593 predicted genes. Several metabolic clusters were identified for the synthesis of polyketide synthases and non‐ribosomal peptide synthases. The synteny of one of the latter clusters facilitating the synthesis of destruxin B was determined with the presence/absence polymorphism of *DtxS1* and *DtxS3* genes being critical for toxin biosynthesis.^14^

A transcriptomic analysis of the *A. brassicae* isolate J3^15^ highlighted the importance of toxin and cell wall metabolism for pathogenicity.^16^ Pathogen genes involved in toxin and secondary metabolism were differentially expressed at 2 days post‐inoculation (dpi) of *B. juncea* cv. Varuna. At 4 dpi, a shift in gene expression occurs with cell‐wall polysaccharide catabolism, presumably contributing to the necrotrophic phase of pathogenesis. In contrast, little is known about host transcriptomics in *B. juncea* after *A. brassicae* inoculation.

The aim of this study was to better understand grey leaf spot disease using genomics and transcriptomics. A new high‐quality genome sequence of a European *A. brassicae* isolate (AA1/5) was generated (based on long‐read Pac Bio and short‐read Illumina sequencing) that was compared to the published genome sequence of the above‐mentioned Indian isolate. Two cultivars of *B. juncea*, Pusa Jaikisan and Sej‐2(2),^17^ were challenged with the pathogen isolate AA1/5. Pathogen and host gene transcriptomics were investigated, and new insights were reported about this pathosystem.

## 2 MATERIALS AND METHODS

### 2.1 Pathogen isolate culture conditions and DNA extraction

The *A. brassicae* isolate AA1/5 was collected from seed of *Brassica napus* grown at Rothamsted Research, UK (WGS85 lat., log. 51.81021, −0.37500), harvested in July 1987. This isolate was used for genome sequencing and pathogen inoculation experiments. For DNA extraction from culture, the isolate was grown on sterile cellulose discs (A.A. Packaging Ltd, Preston, UK) overlaid above 3% potato dextrose agar (PDA; Sigma Aldrich, Merck, Darmstadt, Germany) for 7 days at 18 °C in the dark. Mycelium was harvested by scraping fungal growth from the actively growing edge of the colony. The harvested material was placed into a 2‐mL screw‐top tube and immediately ‘snap frozen’ by placing the tube into liquid nitrogen. It was then lyophilised using a Mechatech LSBC55 LyoDry Compact Freeze Dryer (Bristol, UK) fitted to an RV5 pump.

Subsequently, genomic DNA was extracted using a MasterPure Yeast DNA Kit (Epicentre, Madison, Wisconsin, USA) according to the manufacturer's instructions. Extracted DNA was quantified with a Qubit® 2.0 Fluorometer (Thermo Fisher Scientific Inc., Birmingham, UK) and sent for sequencing to the Beijing Novogene Bioinformatics Technology Co., Ltd.

### 2.2 Genome sequencing, assembly and annotation

#### 2.2.1 Library construction

Sequencing of *A. brassicae* isolate AA1/5 utilised the PacBio Sequel platform and Illumina NovaSeq PE150. The PacBio Sequel platform library for single‐molecule real‐time (SMRT) sequencing was constructed with an insert size of 20 kb using the SMRTbell™ Template Kit (version 1.0). Library quality was assessed using a Qubit® 2.0 Fluorometer (Thermo Fisher Scientific Inc.) with insert fragment size determined using an Agilent 2100 Bioanalyzer (Agilent Technologies, Stockport, UK). For the Illumina NovaSeq platform, 1 μg of total sample DNA was used as input material. Sequencing libraries were generated using NEBNext® Ultra™ DNA Library Prep Kit for Illumina (NEB, Ipswich, USA) following the manufacturer's recommendations. Briefly, the DNA sample was fragmented by sonication to a size of 350 bp, and DNA fragments were end‐polished, A‐tailed, and ligated with full‐length adaptors for Illumina sequencing with further PCR amplification. Lastly, PCR products were purified (AMPure XP system, Beckman Coulter, Amersham, UK) and libraries were analysed for size distribution by an Agilent 2100 Bioanalyzer and quantified via real‐time PCR.

#### 2.2.2 Genome assembly

To ensure the accuracy of subsequent downstream analyses, small (<500 bp) low‐quality PacBio reads were removed. Using the automatic error correction function of the SMRT portal, long reads (>6000 bp) were selected as the seed sequence and shorter reads aligned to the seed sequence using Blasr software, so that the accuracy of the seed sequence could be further improved. This initial assembly was corrected using the variant caller module of the SMRT Link software (v5.0.1); the arrow algorithm was used to correct and count variant sites in the preliminary assembly results. N50 was calculated by sorting of contigs in descending size order, followed by summing the contigs from largest to smallest until the total represented 50% of the total assembly length; the N50 represented the length of the shortest contig in this 50% sum.

#### 2.2.3 Genome component and gene function predictions

Genome component predictions, including coding genes, were made using Augustus (v. 2.7). For gene function predictions, seven databases were used: Gene Ontology (GO), Kyoto Encyclopedia of Genes and Genomes (KEGG), Clusters of Orthologous Groups (KOG), Non‐Redundant Protein Database (NR), Transporter Classification Database (TCDB), cytochrome P450, and Swiss‐Prot. Diamond software was used to align the protein sequences of each predicted gene against each of the seven databases via BLAST searches (E‐value <1 × e^−5^, minimal alignment length percentage larger than 40%). Carbohydrate‐active enzymes were predicted using the Carbohydrate‐Active enZYmes (CAZy) database. The secretory proteins were predicted by the SignalP (version 4.1) database. Secondary metabolism gene clusters were analysed using antiSMASH (version 4.0.2). The Pathogen Host Interactions (PHI‐base, version 4.9) database was screened for genes experimentally verified to influence host‐pathogen outcomes. Interspersed repetitive sequences were predicted using RepeatMasker (http://www.repeatmasker.org/; v. open‐4.0.5); tandem repeats were analysed by the Tandem Repeats Finder (v. 4.07b).

### 2.3 Phylogenetic analysis of *Alternaria* spp.

Sequences from four genes were used in the present study: internal transcribed spacer (ITS, 564 bp), *Alternaria* allergen gene (*Alt a1*, 464 bp), glyceraldehyde‐3‐phosphate dehydrogenase (*GADPH*, 585 bp), and plasma membrane ATPase (*pmATP*, 1268 bp).^18^ This gave a total concatenated length in aligned sequences of 2874 bp (all figures given including gaps). The species/sequences used in the phylogenetic analysis are given in Supporting Information, Table S1 and represent *Alternaria* species most commonly associated with *Brassica* spp. plus *A. brassicae* reference isolates from India (J3) and the USA (EGS 38032). Sequences from UK *A. brassicae* isolate AA1/5 were sourced from the genome reported in this study. The alignment was loaded into Geneious (v. 10.2.3, Biomatters Ltd, Auckland, New Zealand) and underwent alignment with MUSCLE. Bayesian analysis was carried out using version 3.2.6. of MrBayes to which the GTR+G model (the model used in Blagojević *et al*.^18^; model appropriateness reconfirmed using jModelTest v. 2.1.10). Four Markov Chain Monte Carlo (MCMC) chains were run for 1 000 000 generations (with a 25% burn‐in length) at which point convergence was clear (standard deviation of split frequencies <0.01). Tree sampling frequency was set to every 500 generations with diagnostics calculated every 5000 generations. In the analyses, the more distantly related *Stemphylium callistephi* was included as an out‐group. All Bayesian posterior probability values were >0.70 and are given in the tree. The final consensus phylogram was constructed in TreeView (v.1.6.6); Bayesian posterior probability values were all >0.70 and are shown in the tree.

### 2.4 Plant material, growth, inoculation, and sample collection

Seeds of *B. juncea* cultivars Sej‐2 (2) and Pusa Jaikisan were sown in plug cells and when two true leaves were unfolded, the plants were potted on into 20‐cm plastic pots. The plants were grown to the flowering growth stage in a heated glasshouse at 20 °C for 16 h during the day (04:00–20:00) and at 16 °C at night (Fig. 1(A)). Supplementary lighting was used during the day period if natural light fell below 250 W m^−2^.

**Figure 1:**
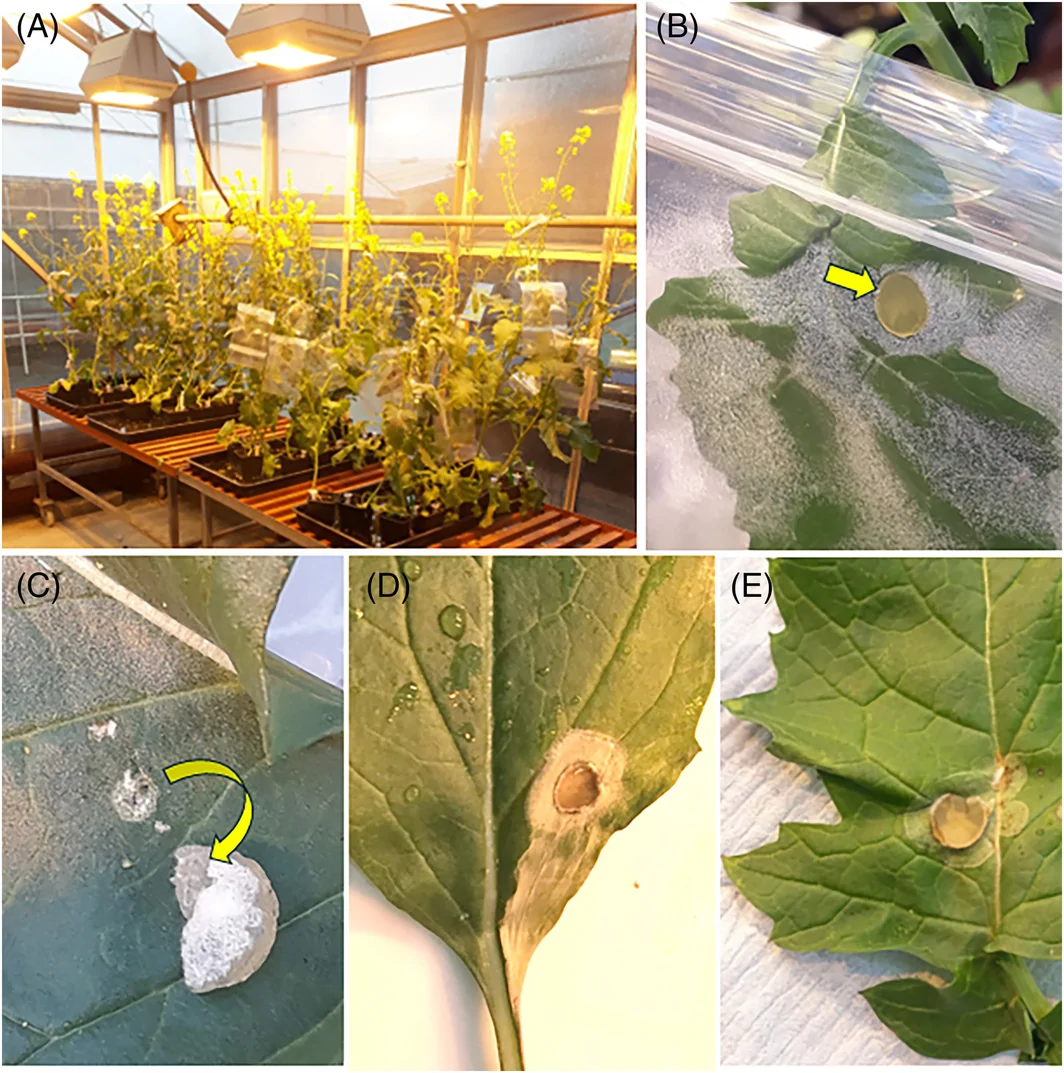
(A) Plant growth conditions at inoculation. (B) *Alternaria brassicae* mycelial plug inoculated onto leaf. (C) Example of removal of mycelial plug at 1‐day post‐inoculation (dpi) to show initial infection of leaf. (D) Example of infection on Sej‐2(2) at 4 dpi. (E) Example of infection on Pusa Jaikisan at 4 dpi. A 20 mm × 20 mm leaf section, centred on the inoculation position was sampled for RNA extraction with the original inoculum removed in each case.

Leaves were inoculated under glasshouse conditions (October 2020) by placing a 6‐mm disc of mycelium of isolate AA1/5 growing directly on PDA (without cellulose discs that were used in section 2.1) mycelium downwards in contact with adaxial leaf laminae. Inoculated leaves were then enclosed in a zip‐lock polythene bag to maintain high humidity and incubated until sampled (Fig. 1(B)). Samples were collected at four time points: day 0 (uninoculated control), day 1, day 2, and day 4. There were five replicates per time point and cultivar. In addition, three replicates of the pathogen's mycelium alone were harvested from cellulose discs above PDA (described earlier); only two of the samples fulfilled RNA sequencing (RNA‐seq) quality requirements, and a duplicate was therefore used. Leaf samples were taken by detaching the inoculated leaf and cutting a 20 × 20 mm square of leaf around the inoculation point (or a representative section of leaf for the uninoculated controls). The original disc of mycelium and agar was carefully removed, and the leaf section was placed into a 15‐mL centrifuge tube, which was immediately snap frozen in liquid nitrogen. Samples were stored at −80 °C ahead of RNA extraction.

### 2.5 RNA extraction, sequencing, and RNA‐seq data analysis

Sample preparation for RNA‐seq analysis was essentially done as previously reported.^19^ Leaf samples were taken from the −80 °C freezer and briefly transported from Rothamsted Research to the nearby University of Hertfordshire on dry ice prior to extraction of RNA using an E.Z.N.A. Plant RNA Kit (Omega Bio‐tek, London, UK).^19^ RNA quantity was assessed using a Nanodrop ND‐1000 spectrophotometer (Thermo Fisher Scientific Inc.) and a Invitrogen™ Qubit® 3.0 Fluorometer (Thermo Fisher Scientific Inc.). A TapeStation 4150 (Agilent Technologies) was used to assess RNA quality prior to shipment to GENEWIZ Germany GmbH (AZENTA Life Sciences, Leipzig, Germany) for library preparation and RNA‐seq. While all samples were replicated in duplicates, most were in triplicates. RNA quality control checks and sequence analyses eliminated specific samples.

#### 2.5.1 RNA‐seq data analysis

RNA samples were sent for [poly‐A+ RNA selection] library preparation and sequencing by Azenta‐Genewiz using an Illumina NovaSeq 150 bp paired‐end protocol. The resulting fastq files were checked using FastQC v0.11.9.^20^ Adapters and reads shorter than 80 bp were removed with Trimmomatic v0.39.^21^ HISAT2 v2.2.1^22^ was used to map the reads for each sample to the reference genomes for the host or the pathogen or both. Transcript abundance was estimated using featureCounts v2.0.3^23^ and the transcript abundance tables were analysed using the BioConductor R package DESeq2.^24^

The reference genome used for the host was *B. juncea* cv. tumida T84‐66V2.0 with 100 828 annotated genes.^25^ The reference genome used for the grey leaf spot pathogen was *A. brassicae* isolate AA1/5 with 7228 gene models. The percentage of reads mapped to each of the organisms totalled 93% across all samples, with fewer than 0.5% of reads mapped to two organisms. On average, across all leaf samples, 82% of reads mapped to the host *B. juncea*. On average across all inoculated leaves, 16% of reads mapped to the pathogen *A. brassicae*. In the mycelium samples, mapping rates to *A. brassicae* were about 88%. Mapping data suggested that one of the *B. juncea* cv. Pusa Jaikisan samples at 1 dpi with *A. brassicae* was misidentified and therefore it was excluded from the analysis.

GO enrichment analysis was done in R using the topGO package as previously described.^19^ The top 20 GO terms were compiled and the top five significant nodes were visualised to better understand the data. The ComplexHeatmap package^26^ was used to visualise differentially expressed genes (DEGs). The area under the gene expression curve in analogy to the area under the disease progress curve was calculated^27^ to highlight annotated genes with the largest differences in gene expression based on quantile calculations.

### 2.6 Quantitative reverse transcriptase‐polymerase chain reaction

For analysis of *PR‐4*, *PR‐5*, and actin gene expression, primers were designed using the Geneious Prime software and checked for their specificity against the *B. juncea* genome. Primers were first tested using conventional reverse transcriptase‐polymerase chain reaction (RT‐PCR) (Table 1). A Stratagene Mx3000P cycler (Agilent Technologies, Santa Clara, CA, USA) was used for quantitative RT‐PCR and data were analysed with the MxPro v4.10 software. A SYBR green master mix (Agilent Technologies) was used for a two‐step PCR with an initial denaturation at 95 °C for 30 min and 40 cycles of 95 °C denaturation and 60 °C for 20 s each. The melt curve analysis was done at default settings. Standard curves were produced for each of the five tested genes; each run included standard curves of the test gene and the actin reference gene. Gene expression was calculated using normalised relative quantities^28^ from the slopes of at least two standard curves divided by the mean of the positive, constitutively expressed, control actin to give a normalised count. The log2 was then calculated and used for plotting data. Three biological replicates were used per sample and two technical repeats were done.

**Table 1:** Primers used in this study

Gene IDs	Sequence 5′ to 3’	Closest hit
*PR‐5*/BjuVA06G29420	CGGACAGGACTTCTACGACG CGCTCTTACATGCCACAACG	AT1G19320
		Pathogenesis‐related thaumatin superfamily protein
*PR‐5*/BjuVB04G25850	GGTGGGTTTCTCTTAGCCCC	AT1G19320
	GTTGCCGATCGTGAATTCGG	
		Pathogenesis‐related thaumatin superfamily protein
*PR‐4*/BjuVA03G29410	CCAAAACAGCCGCTCAATCC	AT3G04720
		HEVEIN‐LIKE, pathogenesis‐related
	TTGGTTCCCATCCCATGTCG	
*PR‐4*/BjuVB07G27970	CGCTCAGTCCGCTAACGTAA	AT3G04720
	ACTCAAGCGGTTGGTTTCCA	
		HEVEIN‐LIKE, pathogenesis‐related
BjuVB08G57360	ATCACCATCGGAGCTGAAAG	*ACT7* (AT5G09810)
	GAACCACCACTGAGGACGAT	

BLAST searches were done against the TAIR database.

For statistical analysis, the four *PR* genes were tested separately for normality (Shapiro–Wilk <0.05). As the data for the four genes were not normally distributed, a Kruskal–Wallis test was performed to assess significance between cultivars (*P* > 0.05) and time (*P* < 0.0001). Furthermore, a Dunn's multiple comparisons analysis was used to determine the statistical significance between individual time points.

## 3 RESULTS

### 3.1 Genome assembly and characterisation

The optimised genome assembly of UK *A*. *brassicae* isolate AA1/5 comprised a total length of ~32.6 M bp, assembled to 25 contigs with an N50 of 3.4 M bp and 50.8% GC content (Table 2). Gene prediction, using Augustus (v. 2.7), identified 7228 genes, with an average size of 1438 bp and in total representing ~32% of the genome (Supporting Information, Table S2). In total, ~2.76% of the genome was predicted to be interspersed repetitive sequences, with the majority being long terminal repeats (~1.11%) or DNA transposons (~1.56%); in addition, ~0.5% of the genome was predicted to be tandem repeats, including ~0.32% minisatellite DNA and 0.1% microsatellite DNA.

**Table 2:** Summary of genome assembly and comparison with another *Alternaria brassicae* genome sequence

Parameters	Assembly stats	Assembly stats
Isolate name	AA1/5	J3*
Country of origin	U.K.	India
Sequencing technology	PacBio, Illumina	Oxford Nanopore
Number of contigs	25	17
Number of contigs (>10 000 bp)	25	17
Maximum length (bp)	8 390 722	7 015 266
N50 length (bp)	3 395 661	2 988 132
Total length (bp)	32 642 406	34 141 106
GC (%)	50.8	50.7

* Rajarammohan *et al*.^40^

Gene function predictions, based on the GO, KEGG, KOG, NR, TCDB, P450 and Swiss‐Prot databases, are given in Supporting Information, Table S2. For secreted protein prediction, 632 proteins were identified with a signal peptide using SignalP (Supporting Information, Table S3). Twenty secondary metabolite gene clusters were predicted via antiSMASH on eight contigs, namely five terpene clusters (23 genes), five T1PKS clusters (31 genes), one NRPS, T1PKS cluster (17 genes), five NRPS‐like clusters (48 genes), and four NRPS clusters (30 genes) (Supporting Information, Table S4). A local BLAST identified homologs of *AbrePsy1* (A3079) and the neighbouring *AbreAtr1* (A3080) gene on the NRPS cluster 9; the former encodes a large protein that catalyses destruxin B biosynthesis and the latter an ABC transporter.^29^ Both genes were well expressed during plant colonisation and in culture.

Screening against the CAZy database revealed several predicted genes relating to carbohydrate enzymes including glycoside hydrolases (226 genes), glycosyl transferases (82 genes), auxiliary activities (71 genes), carbohydrate modules (56 genes), carbohydrate esterases (37 genes), and polysaccharide lyases (19 genes) (Supporting Information, Table S5). Lastly, screening against the PHI‐base database identified several genes that have been demonstrated experimentally to affect host–pathogen interactions, with phenotypes including chemical resistance (three genes), effectors (seven genes), increased virulence (27 genes), lethality (69 genes), loss of pathogenicity (107 genes), reduced virulence (472 genes), but also unaffected pathogenicity (367 genes) (Supporting Information, Table S6). Among the effector genes were four cell death inducers, *MoCDIP1* (A4334) and *MoCDIP4* (A1841, A2938, A4096), as well as A1242, a *PsXEG1* homolog involved in xyloglucan catabolism. Out of a total of 1127 genes, 75 genes had multiple hits on PHI‐base with contrasting phenotype outcomes; these were therefore defined as not assigned (Supporting Information, Table S6).

### 3.2 Phylogenetic analysis of *Alternaria* spp.

Phylogenetic analyses using concatenated sequences of the ITS, *Alt a1*, *GAPDH*, and *pmATP* genes confirmed the species identity of UK isolate AA1/5 as *A. brassicae*, being clearly distinct from other related *Alternaria* spp. isolated from brassicas (Fig. 2). Sequence comparisons of the newly sequenced UK isolate AA1/5 with reference Indian J3 and USA EGS 38032 *A. brassicae* isolates revealed 100% identity for the *ITS, Alt a1*, and *GADPH* genes. For the *pmATP* gene, the UK isolate exhibited 99.9% similarity [one single nucleotide polymorphism (SNP) difference] with reference Indian isolate J3, and 99.7% similarity (two SNPs different) with reference USA isolate EGS 38032. No evidence for structuring of *A. brassicae* isolates was thus obtained, based on the four genes sequenced in this study, despite the isolates used originating from three separate continents (Europe, Asia, North America).

**Figure 2:**
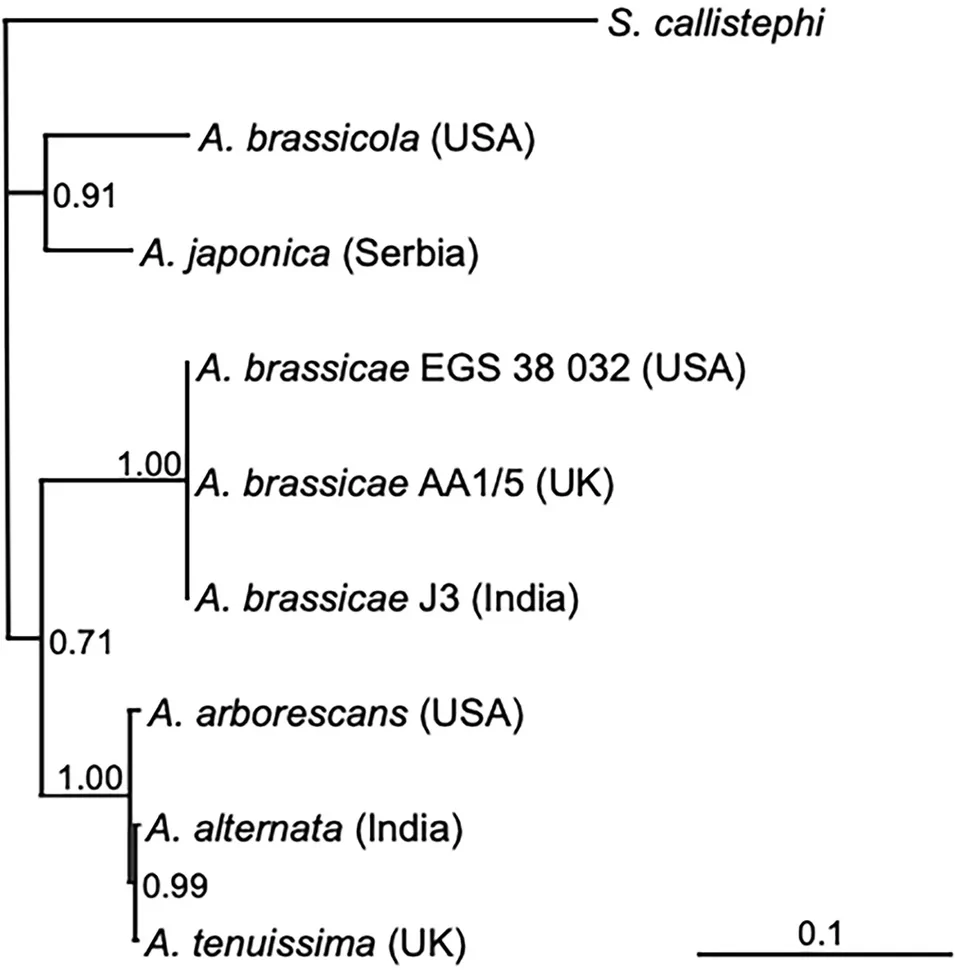
Consensus Bayesian phylogenetic tree (1 000 000 generations, 25% burn in) based on concatenated sequences from four genes (ITS, *Alt a1*, *GAPDH*, *pmATP*) of *Alternaria* species isolated from *Brassica* spp. Sequences of *A. brassicae* isolate AA1/5 were obtained from the genome sequence reported in the present study. *Stemphylium callistephi* was selected as the outgroup for this tree. Bayesian posterior probability values are indicated at nodes. Scale bar indicates substitutions/site.

### 3.3 Inoculation of *B. juncea* with *A. brassicae* for transcriptomic profiling

Initial symptoms were already visible at 1 dpi with the *A. brassicae* isolate when removing the agar plug from the leaf surface (Fig. 1(C)). Symptoms were more severe at 4 dpi with the pathogen when cultivars Sej‐2(2) (Fig. 1(D)) and Pusa Jaikisan (Fig. 1(E)) were compared. Collective visual inspection of the symptoms from all inoculations suggested that cv. Pusa Jaikisan was more susceptible than cv. Sej‐2(2).

### 3.4 Gene expression during foliar pathogen infection

Transcriptomics identified a total of 78 299 plant genes from infected or uninfected leaves of *B. juncea* cultivars when comparing to the annotated *B. juncea* cv. tumida genome, suggesting that 78% of the annotated genes were expressed under the conditions in which the cultivars were analysed. A total of 7202 expressed fungal genes were identified using comprehensive data from nutrient agar‐grown mycelia and pathogen‐infected leaves in comparison to the in‐house *A. brassicae* isolate AA1/5 genome data. This demonstrates that only 26 of the fungal genes were not expressed under the experimental conditions tested. Differential gene expression analysis identified cohorts of pathogen and host genes that specifically responded to the foliar infection process under investigation.

### 3.5 Infection alters expression of pathogen genes involved in carbohydrate metabolism and endocytosis

Principal component analysis (PCA) showed significant changes in *A. brassicae* gene expression after inoculation of *B. juncea* leaves at the different time points after infection (Fig. 3). Gene expression of *in vitro* grown *A. brassicae* (0 dpi) clearly differed from fungal gene expression during *in planta* colonisation. The pathogen genes expressed at the different time points were also clearly different. Except for 1 dpi, the effect of host cultivars on pathogen gene expression were not readily apparent.

**Figure 3:**
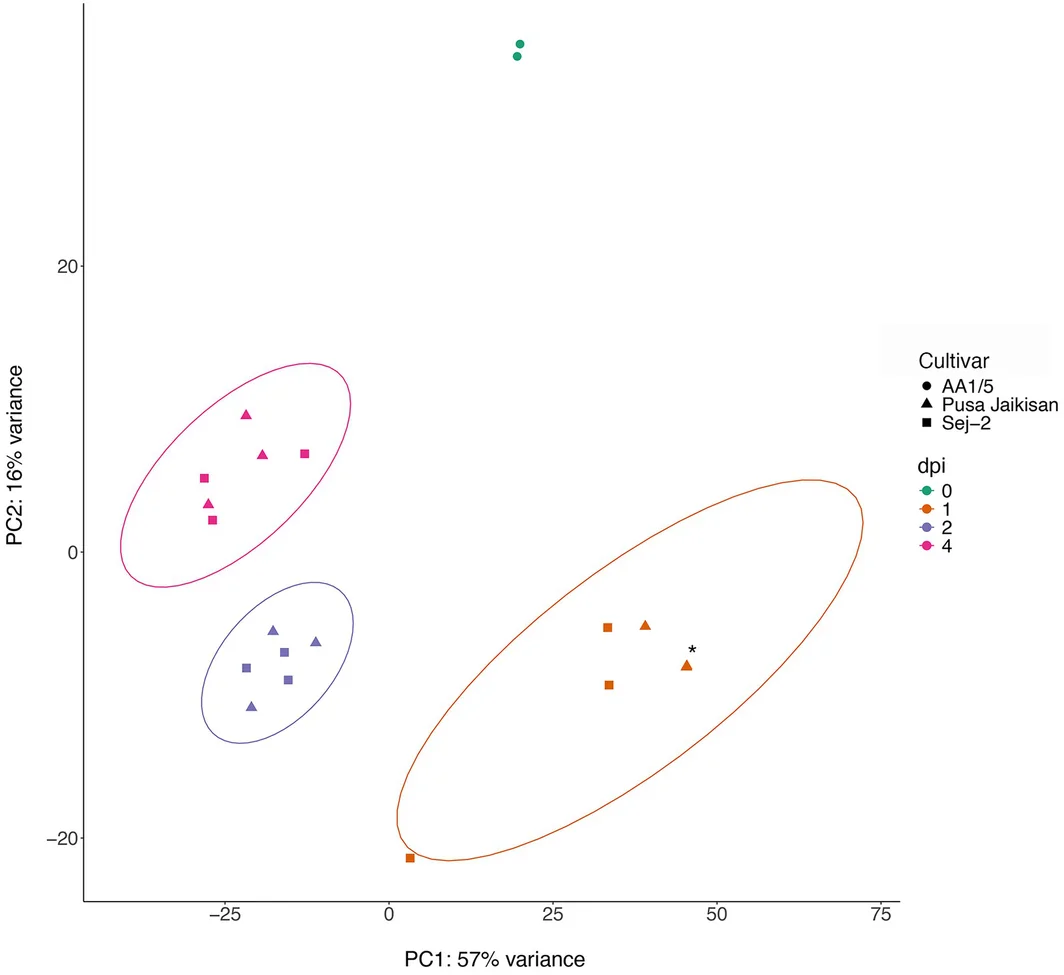
Principal component analysis (PCA) plot of *Alternaria brassicae* gene expression as a function of days post‐inoculation (dpi) and cultivar of *Brassica juncea*. Note that isolate AA1/5, synonymous with 0 dpi, displays gene expression of *in vitro* grown *A. brassicae*. Pusa Jaikisan and Sej‐2 (2) were *B. juncea* cultivars used in this study. Multivariate normal distribution was displayed by ellipse. All samples were in triplicate except for *in vitro* grown mycelia, which were in duplicate as stated in Materials and Methods. The asterisk indicates a triangle that represents two overlapping samples of Pusa Jaikisan at 1 dpi.

GO enrichment analysis was done to identify terms of interest (Table 3). When comparing *in vitro versus in planta* expression, all four Arp2/3 complex proteins (A3302, A5206, A5507, A6657) were co‐ordinately down‐regulated *in planta*, albeit at ~log2‐fold, suggesting down‐regulation of endocytosis.^30^ Among the 35 most highly expressed genes in planta were glucanases (A2042, A3922), pectin lyases (A0986, A6895) and necrosis‐and ethylene‐inducing protein 1 (*NEP1*, A4220), suggesting involvement of cell wall degradation and induction of host cell death (Supporting Information, Table S7). Local BLAST identified a putative homolog of the ABR‐toxin (A1662), encoding a trypsin precursor; the predicted molecular mass (23 kDa) and pI (8.3) of the mature peptide were not incompatible with it being the ABR‐toxin.^3^ A1662 was expressed more strongly *in vitro* than *in planta* (Supporting Information, Table S7).

**Table 3:** Enrichment analysis of Gene Ontology terms using differentially expressed genes in *Alternaria brassicae*

	Number of genes*	BP	CC	MF
*In vitro vs in planta*	1133	Electron transport chain (6/9)^†^, protein glycosylation (9/19)	Membrane (122/536), mitochondrial respirasome (5/5), **Arp2/3 protein complex (4/4)** ^‡^	Inorganic cation transmembrane transporter activity (38/119), oxidoreduction‐driven active transmembrane transporter activity (13/23), carbon oxygen lyase activity (12/24), electron transfer activity (16/43)
D2 *vs* D1	392	Transmembrane transport (38/354), **carbohydrate metabolic process (28/256)**, nitrate assimilation (3/6)	Host cell cytoplasm (1/1), Atg1/ULK1 kinase complex (1/1), acetyl‐CoA carboxylase complex (1/1), photosystem I reaction centre (1/1)	Hydrolase activity, hydrolysing O‐glycosyl compounds (28/157), oxidoreductase activity (70/643), oxidoreductase activity, acting on paired donors, with incorporation or reduction of molecular oxygen (17/91)
D4 *vs* D1	1330	Ribosome biogenesis (19/45), alpha‐amino acid catabolic process (8/13)	**Membrane coat (12/41)**, nucleolus (5/9), HDA1 complex (4/6)	Oxidoreductase activity (160/643), dioxygenase activity (10/20), catalytic activity (535/2486), 3‐hydroxyisobutyryl‐CoA hydrolase activity (6/9), NAD binding (27/79)
D4 *vs* D2	477	Potassium ion transport (5/30), steroid biosynthetic process (5/32), cristae formation (2/4)	Mitochondrion (9/77), MICOS complex (2/4), membrane coat (5/41)	Catalytic activity (126/2486), Carbon‐sulfur lyase activity (4/11), acireductone dioxygenase [iron(II)‐requiring] activity (2/3), 3‐beta‐hydroxy‐Delta5‐steroid dehydrogenase (NAD+) activity (5/31)
Cv. diff.	203	Methionine metabolic process (2/3)	Preribosome (4/34), nuclear protein containing complex (9/189), mitotic spindle (2/11)	Catalytic activity (94/2478), 5‐methyltetrahydropteroyltriglutamate‐homocysteine S‐methyltransferase activity (2/3), thiamine pyrophosphate binding (3/12)
Cv. × D2	70	Methionine biosynthetic process (2/3)	Preribosome, small subunit precursor (1/1), nematocyst (1/2), cytoplasm (6/358)	Oxidoreductase activity (16), S‐methyltransferase activity (2)
Cv. × D4	21	rRNA processing (2/29), sulfur amino acid biosynthetic process (1/3)	Preribosome, small subunit precursor (1/1), RNA polymerase III complex (1/9)	Structural constituent of cuticle (1, 2), Uroporphyrinogen decarboxylase activity (1/2), 5‐methyltetrahydropteroyltriglutamate‐homocysteine S‐methyltransferase activity (1/3)

* Five main effects and an interaction term are shown using normalised expression with the vsd function of the DESeq2 package; Padj <0.01.

^†^ Observed *vs* expected values are shown relative to all the expressed genes identified, *P* < 0.05.

^‡^ Bold text is discussed in the Results section.

A comparison of different infection stages revealed that the carbohydrate metabolic process was significantly enriched when comparing 1 and 2 dpi. Three glucanases were induced at 2 dpi relative to 1 dpi, including the above‐mentioned endoglucanase A2042 being induced but exoglucanase A3922 less so (Supporting Information, Table S8). Comparison of 1 and 4 dpi identified membrane coat components as enriched. Expression of genes encoding clathrin adaptor complexes (A3106, A6885) and clathrin heavy chain (A6667) was repressed during later stages of infection, implicating down‐regulation of endocytosis during infection (Supporting Information, Table S9).

### 3.6 Host gene expression after *A. brassicae* inoculation

PCA showed separate clusters for the infection time points *versus* the uninoculated control at 0 dpi (Supporting Information, Fig. S1). There were also distinct clusters between the two cultivars Pusa Jaikisan and Sej‐2(2), indicating differences in gene expression between these different cultivars in response to *A. brassicae* infection (Fig. 4).

**Figure 4:**
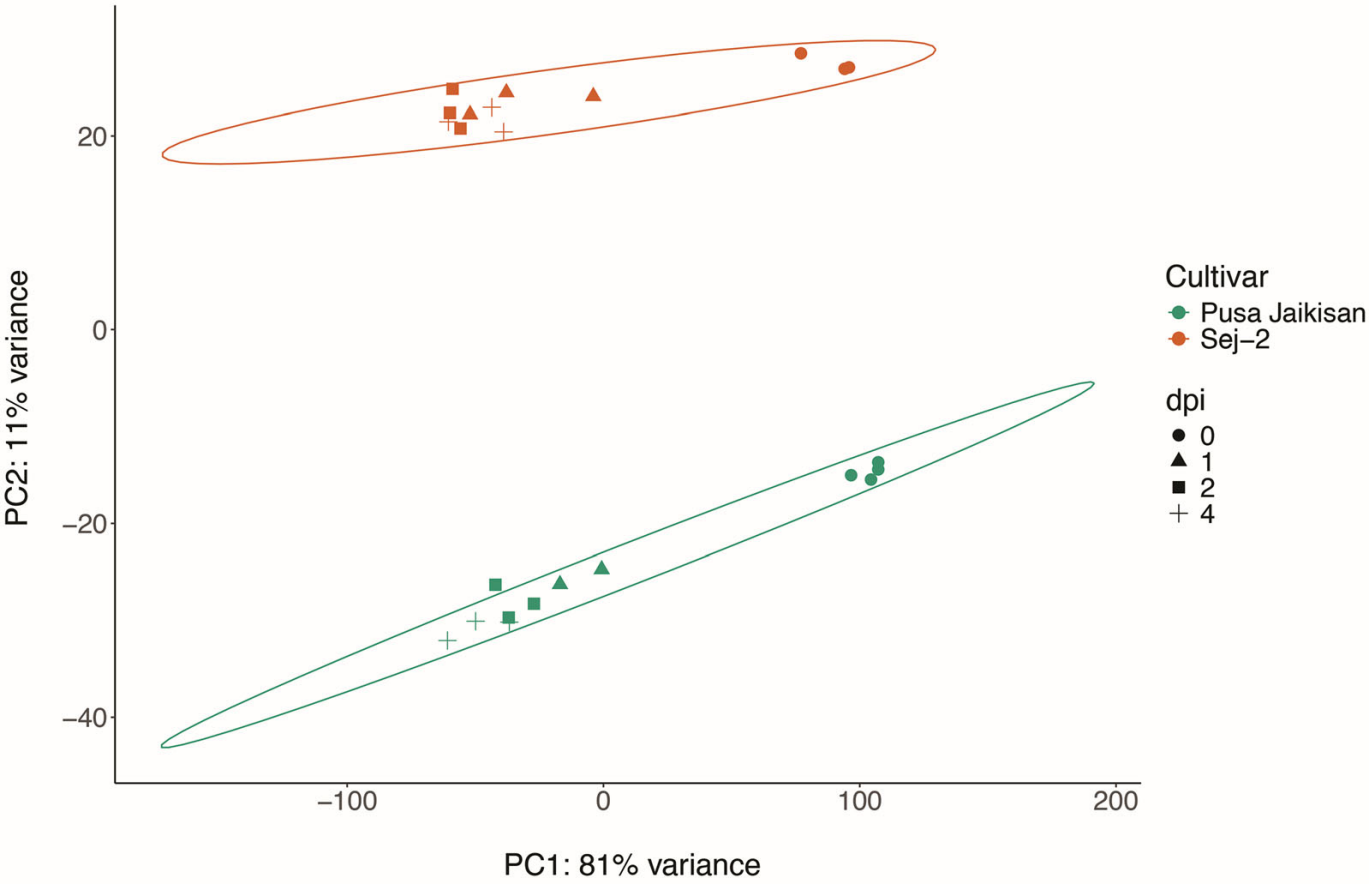
Principal component analysis (PCA) plot of *Brassica juncea* gene expression. Cultivars Pusa Jaikisan and Sej‐2(2) were uninoculated, 0 days post‐ inoculation (dpi), or inoculated with *Alternaria brassicae* and leaf samples harvested at different time points (dpi). The plot displays variance in gene expression as a function of time (dpi) and cultivar. The ellipse displays multivariate normal distribution by cultivar. All samples were in triplicate except for samples at 0 and 1 dpi in cultivar Pusa Jaikisan, which represented a quadruplicate and duplicate, respectively.

GO enrichment analysis of the different time points after inoculation identified photosystems (PS) and particularly PS I as a target of *A. brassicae* infection (Table 4). More PS genes were significantly enriched at 2 dpi than at the other time points. Heatmap analysis demonstrated a difference between the two cultivars. Whereas the expression of PS genes continuously declined in Pusa Jaikisan over the entire time course of 4 days, PS gene expression in Sej‐2(2) reached a minimum at 2 dpi with a recovery and increase of PS gene expression again at 4 dpi (Fig. 5(A)). Despite this recovery of PS gene expression at 4 dpi, expression of most PS genes at 2 dpi in Sej‐2(2) was lower than in Pusa Jaikisan at any time. Among the 77 identified *B. juncea* genes were 21 *Arabidopsis thaliana* homologs, 12 of which were related to PS I^31^ and eight of which belonged to PS II.^32^ In addition, expression of the transcription factor *IAA11* was reduced by *A. brassicae* infection (Supporting Information, Fig. S2). It is tempting to speculate that down‐regulation of *PsbO‐1*, *PsaF* and *IAA11* in particular could contribute to reactive oxygen species production.^33^

**Table 4:** Enrichment analysis of Gene Ontology terms using differentially expressed genes in *Brassica juncea*

	Number of genes*	BP	CC	MF
D1 *vs* D0	14 834	Purine nucleotide metabolic process (31/65)	Thylakoid (67/120), **photosystem I (31/49)**	Structural constituent of ribosome (434/940), oxidoreductase activity (610/2117)
D2 *vs* D0	21 869	ATP biosynthetic process (28/34)	**Photosystem (77/104)**, photosystem I (44/49)	Catalytic activity (4273/11468), oxidoreductase activity (907/2117)
D4 *vs* D0	19 712	**Carbohydrate metabolic process (82/195)**, purine ribonucleotide metabolic process (34/65)	Membrane protein complex (130/255), **photosystem I (34/49)**	Catalytic activity (3645/11468), oxidoreductase activity (712/2117)
D1 specific	1531	Ribosome biogenesis (9/83), mitochondrial respiratory chain complex III assembly (2/5)	Cytoplasm (21/552), mitochondrial matrix (4/28), signal recognition particle (2/8)	Structural constituent of ribosome (256/940)
D2 specific	4698	Nitrogen compound transport (53/382), protein transport (45/329)	Proteasome complex(15/72), proteasome core complex (14/67)	Oxidoreductase activity (197/2117), 4 iron, 4 sulfur cluster binding (4/4)
D4specific	4444	Intracellular transport (45/313), Intracellular protein transport (36/235)	Plasma membrane protein complex (11/34), **1,3‐beta‐D‐glucan synthase complex (10/30)**	Protein binding (385/5439), adenyl ribonucleotide binding (125/1449)
Cv. diff.	2682	Vesicle mediated transport (9/94), defense response to bacterium (2/3), phospholipid biosynthetic process (6/58)	Ino80 complex (2/10), mitochondrial proton‐transporting ATP synthase complex (2/11)	**Chitinase activity (9/37)**, serine‐type endopeptidase inhibitor activity (3/10), **pectate lyase activity (4/21)**
Cv. × D1	80	Regulation of systemic acquired resistance (1/7), **defence response (2/141)**	Endoplasmic reticulum (1/86), membrane (4/1375)	Chitinase activity (3/37), protein‐disulfide reductase activity (4/161), squalene monooxygenase activity (2/17)
Cv. × D2	154	Regulation of systemic acquired resistance (1/7), **defence response (2/141)**	Endoplasmic reticulum (1/86), membrane (4/1375)	Chitinase activity (3/37), protein‐disulfide reductase activity (4/161), squalene monooxygenase activity (2/17)
Cv. × D4	322	Retrograde transport, endosome to Golgi (1/4), **defence response (3/141)**, regulation of response to biotic stimulus (1/7)	Membrane (9/1375)	Chitinase activity (3/37), catalytic activity (79/11468), methionine adenosyltransferase activity (2/17), antiporter activity (5/196)

Numbers of expected/significantly enriched genes identified are shown in parentheses.

* Five main effects and an interaction term are shown using normalised expression with the vsd function of the DESeq2 package; Padj <0.01.

**Figure 5:**
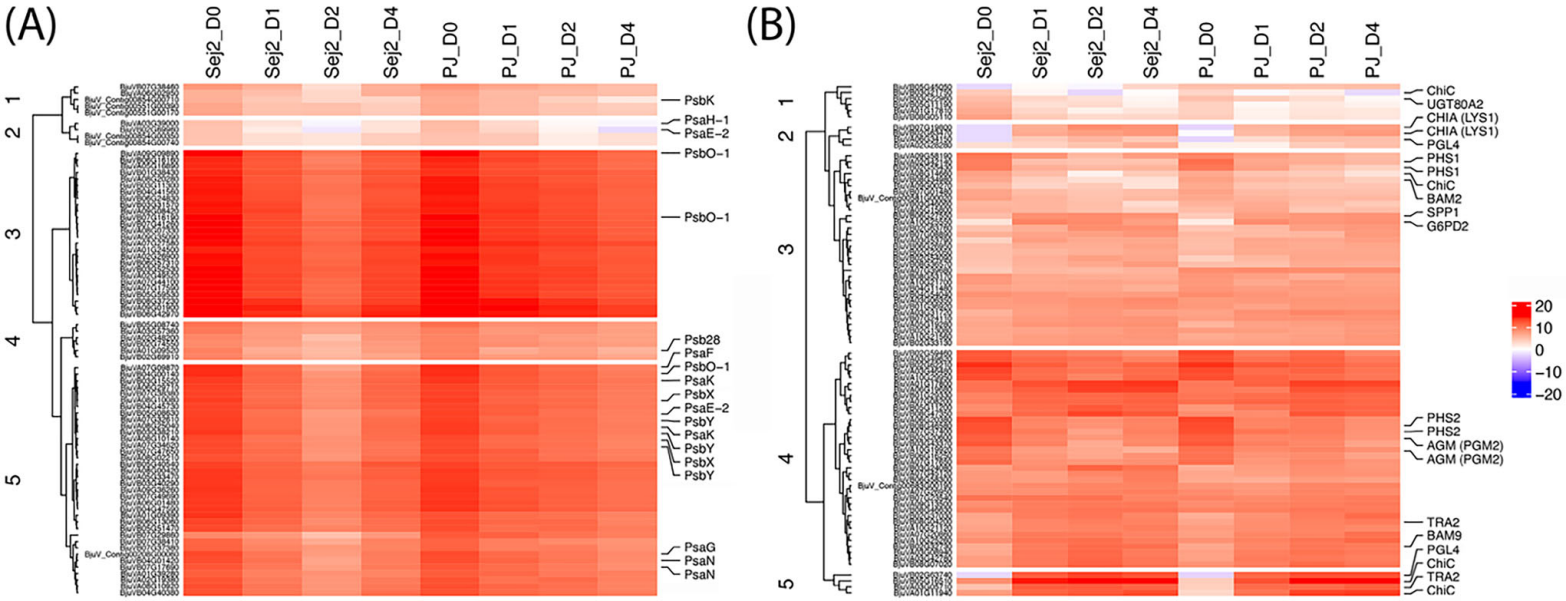
Heatmap of differentially expressed genes (DEGs) of *Brassica juncea*. (A) DEGs enriched in the Cellular Compartment (CC) category ‘photosystem’ when comparing leaves infected with *Alternaria brassicae* at day 2 post‐inoculation (D2) with uninfected (D0) leaves. (B) DEGs enriched in the Biological Process (BP) category ‘carbohydrate metabolic process’ when comparing leaves infected with *Alternaria brassicae* at day 4 post‐inoculation (D4) with uninfected (D0) leaves. Cultivars Sej‐2(2) (Sej2) and Pusa Jaikisan (PJ) were used as hosts. Means of normalised expression values of individual samples are shown according to the colour gradient. Distance trees are shown with five clusters; *B. juncea* gene IDs are shown next to it. Areas under the gene expression curves were calculated for each cultivar, and the gene annotations for the DEGs with the largest temporal expression differences in both cultivars are shown to the right of the heatmap; a quantile >0.75 was used.

Among the Biological Process (BP) category, the carbohydrate metabolic process was significantly enriched when comparing *A. brassicae*‐infected leaves at 4 dpi with uninfected leaves (Table 4). Two immunity‐related genes were pathogen‐induced, *ChiA*, encoding a class III chitinase, also referred to as *Lys1*, and *NHO1*, encoding a glycerol kinase (Fig. 5B and Supporting Information, Fig. S3). Key genes of the oxidative pentose phosphate pathway (OPPP) were induced after *A. brassicae* infection. Glucose‐6‐phosphate dehydrogenase (*G6PD*) catalyses the first committed step in the pathway^34^ and 6‐phosphogluconolactonase (*PGL*) the subsequent reaction.^35^ *G6PD2*, *G6PD6*, *PGL5* and two copies of both *G6PD5* and *PGL4* were induced. Five copies of the pentose phosphate pathway gene transaldolase (*TRA2*) were also induced. Whereas expression of *RPE* genes encoding cytosolic ribulose‐5‐phosphate 3‐epimerase was induced after *A. brassicae* infection, expression of plastid *pRPE* genes was suppressed,^36^ coincident with suppression of PS genes (Fig. 5(A)). Many DEGs related to starch and sucrose metabolism, but no concerted regulation of these genes was apparent.

Expression of 1,3‐beta‐D‐glucan synthases involved in callose biosynthesis was down‐regulated after challenge with *A. brassicae* at 4 dpi when comparing to the other infection time points (Table 4 and Supporting Information, Fig. S4); this was particularly obvious in Sej‐2(2).

### 3.7 Dependence of *A. brassicae*‐induced expression on host cultivar

A total of 2682 DEGs were identified to differ between Sej‐2 (2) and Pusa Jaikisan. While expression of pectate lyases in unchallenged Sej‐2(2) plants was higher than in Pusa Jaikisan, down‐regulation of these genes during infection was stronger in Sej‐2(2), especially at day 2 (Table 4 and Supporting Information, Fig. S5A). The expression profile of chitinases differed between the two cultivars. While class‐IV chitinases were more highly expressed in Sej‐2(2), others, such as *PR‐3* like basic chitinases (At2g43590 ortholog), were more strongly induced in Pusa Jaikisan (Supporting Information, Fig. S5B).

A focus was given to genotypic differences in pathogen‐induced expression at 1 dpi, reasoning that this early stage was less dominated by necrotrophy (Fig. 6). Like the other time points and the cultivar differences in general, the GO‐term defence response featured prominently for the BP category (Table 4). On closer inspection, many of the identified genes were involved in host–pathogen interactions. Areas under the gene expression curve were calculated to better contrast genotypic expression differences. A quantile threshold of 0.8 was set to visualise the genes with the largest expression difference between the two cultivars. This included *RLP35*, *LIK1*, the above‐mentioned *PR‐3* like basic chitinase (Supporting Information, Fig. S5B) and *CRK21*, all of which are strongly induced in Pusa Jaikisan. Notably, *RLP35* and *LIK1* were down‐regulated in Sej‐2(2) (Fig. 6). Conversely, *JOX4* and *DAO2* were more highly induced or expressed in Sej‐2(2) than in Pusa Jaikisan. Other genes identified included *MLO2*, which was suppressed in Pusa Jaikisan but not in Sej‐2(2), and *CYP81F3*, which was more strongly induced in Sej‐2(2) than in Pusa Jaikisan (Fig. S6). These data illustrate that the two cultivars differ in their response to *A. brassicae* infection.

**Figure 6:**
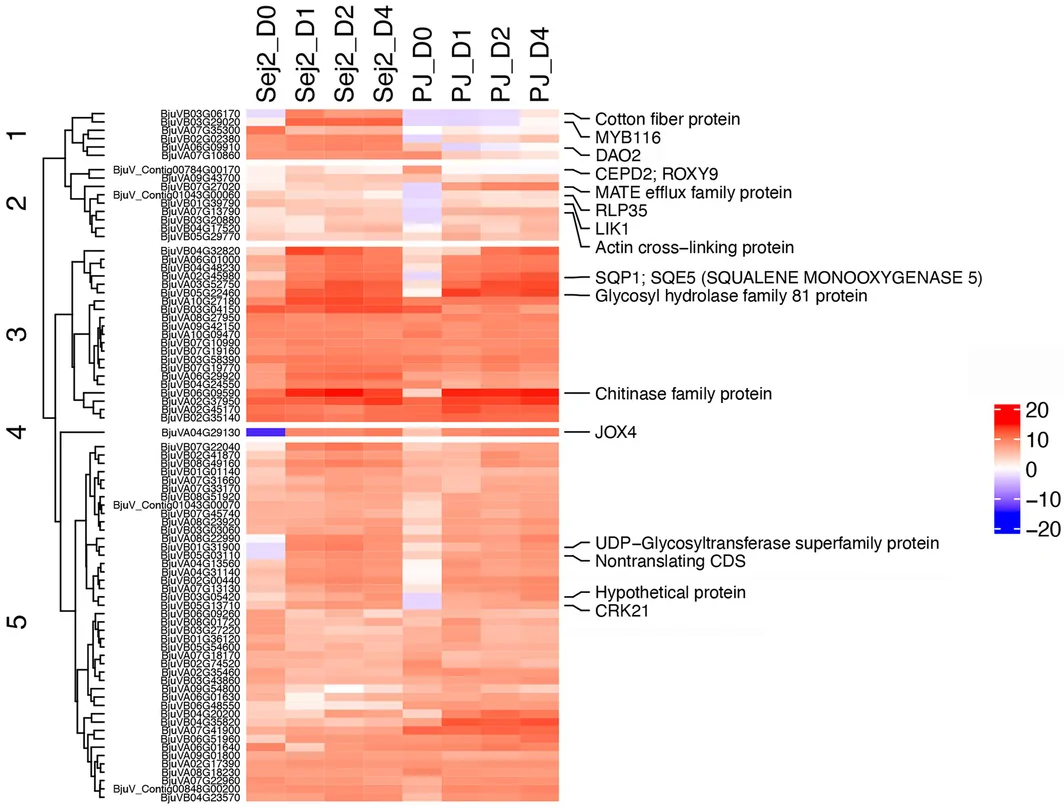
Heatmap of differentially expressed genes (DEGs) in uninfected (D0) or infected leaves of *Brassica juncea* cultivars Sej‐2(2) (Sej2) and Pusa Jaikisan (PJ) with *Alternaria brassicae* at days 1 (D1), 2 (D2) or 4 (D4) post‐inoculation. Means of normalised expression values of individual samples are shown according to the colour gradient. A distance tree is shown with five clusters; *B. juncea* gene IDs are shown next to it. Areas under the gene expression curves were calculated for each of the cultivars, and the gene annotations for the DEGs with the largest differences between the cultivars are shown to the right of the heatmap; a quantile >0.8 was used.

### 3.8 Quantitative PCR analysis of *PR‐4* and *PR‐5* gene expression

To validate the RNA‐seq study, two copies of two pathogenesis‐related (*PR*) genes were studied, *PR‐4* and *PR‐5*. These genes reside on four different chromosomes (chr), namely chrA03 (BjuVA03g29410, *PR‐4*), chrB07 (BjuVB07g27970, *PR‐4*), chrA06 (BjuVA06g29420, *PR‐5*) and chrB04 (BjuVB04g25850, *PR‐5*). The expression of all four genes most dramatically increased during the first day of infection with *A. brassicae* (Fig. 7). There was a further small increase of expression at 2 dpi, but this was not significant for *PR‐5* on chrA06. After this peak of expression at 2 dpi, expression of all *PR‐4* and *PR‐5* genes remained highly induced at the same level at 4 dpi. Unlike the *PR‐5* genes, *PR‐4* genes had a significant increase in expression between 1 and 4 dpi. Small differences in induced expression between *PR‐4* and *PR‐5* were therefore detected. With regards to cultivar, there was no statistical difference across any of the genes studied; the jitter plot, however, does show that the *PR* genes tended to be slightly higher expressed in Sej‐2(2) than in Pusa Jaikisan.

**Figure 7:**
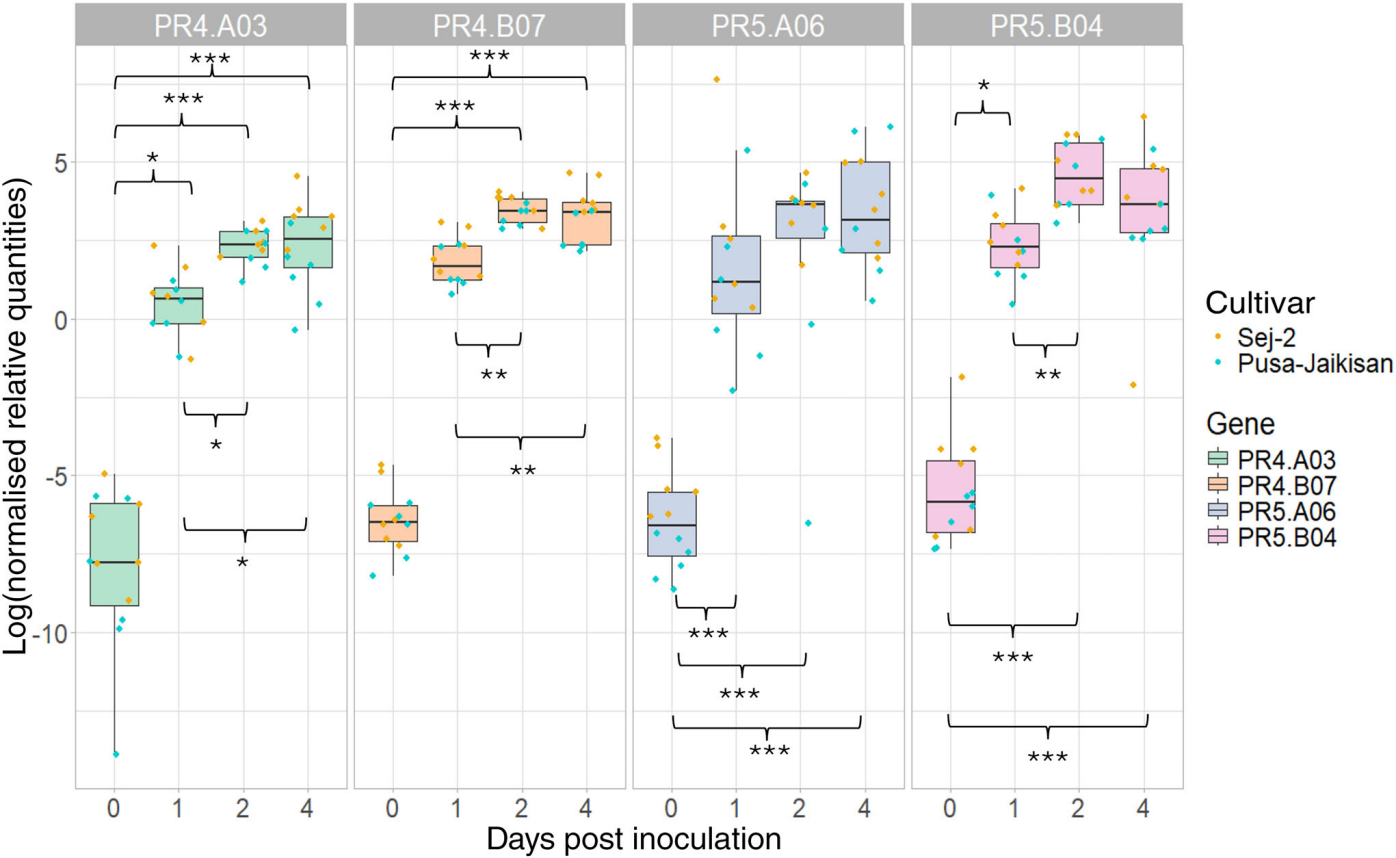
Quantitative PCR of genes of interest from *Brassica juncea* inoculated with *Alternaria brassicae*. RNA was extracted from infected cultivars at 1‐, 2‐ or 4‐days post‐inoculation (dpi) or from uninfected plants (0 dpi). Values were normalised against the constitutive positive control gene encoding actin and log2 transformed. All four pathogenesis‐related (PR) genes showed statistically significant temporal differences (Kruskal–Wallis, *P* < 0.0001), but the effect of the cultivar was not significant. Further analysis was done using Dunn's multiple comparisons test. Three biological replicates and two technical replicates were used. Statistically significant differences are denoted with asterisks (*P < 0.05, **P < 0.01, ***P < 0.001).

## 4 DISCUSSION

### 4.1 *A. brassicae* genome comparisons

This article features the first genome sequence of a UK isolate of *A. brassicae*. PacBio and Illumina sequencing resulted in a total genome size of ~32.6 M bp relative to the size of 34.1 M bp for the Oxford Nanopore‐sequenced J3 isolate from India.^14^ Only 7228 genes were identified in the AA1/5 UK isolate relative to the 11 593 genes identified previously in the J3 isolate and other annotated *Alternaria* genomes. The difference in gene numbers might be due to differences in gene calling methods used. Another contributing factor could be super‐numeric chromosomes, for instance in the Indian isolate. In support, 9.33% of the J3 genome was repetitive, whereas only 2.76% of the AA1/5 genome reported here contained interspersed repetitive sequences. Fewer secreted proteins than in J3 (632 *vs* 1195) were identified in AA1/5, although number of CAZymes was similar (491 *vs* 508). Nevertheless, the total genome sizes of multiple Alternaria genomes were similar.^14^ Among the identified effector genes were cell death inducers, homologous to *Magnaporthe oryzae* CDPI genes,^37^ and an *XEG1* effector gene also known to induce cell death^38^ and interact with corresponding plant immune receptors.^39^

Comparative phylogenetic analyses (based on concatenated sequences from the ITS, *Alt a1*, *GAPDH* and *pmATP* genes) of newly sequenced UK isolate AA1/5 with previously published *A. brassicae* reference isolates from India (J3)^40^ and the USA (EGS 38032)^18^ confirmed the species identity of AA1/5 as *A. brassicae*. Three of the four genes (ITS, *Alt a1*, *GAPDH*) used exhibited 100% similarity; a small number of SNPs (one/two) distinguished the UK and Indian/USA isolates using the longer *pmATP* gene (99.7–99.9% similarity). Thus no clear evidence was obtained, based on phylogenetic analyses undertaken in the present study, for population structuring of *A. brassicae* despite the isolates used originating from three geographically distinct continents (Europe, Asia, North America).

A local BLAST search identified a homolog of *AbrePsy1* (A3079), synonymous with *DtxS1*, involved in destruxin B biosynthesis.^14^, ^29^, ^41^ While A3079 was located next to an ABC transporter (A3080) homologous to *AbreAtr1*,^29^ syntenic relationships with other destruxin B biosynthetic genes like in entomophagic *Metarhizium* species could not be confirmed.^41^ A close comparison of the destruxin B clusters^14^ was not made, although an aldo‐keto reductase (*DtxS3*) was not closely linked to A3079. A putative ABR‐toxin coding sequence (A1662) was also identified using the published N‐terminal amino acid sequence of the mature protein.^3^ The ABR toxin was originally isolated from spore germination fluids, but relative expression during other developmental or infection stages is not known. Here, A1662 was more highly expressed in hyphal tips of *in vitro* cultivated mycelia than during host colonisation. While functional analysis of this putative ABR‐toxin will still be needed, it was shown that genes encoding major pathogenicity determinants of *A. brassicae* contributed to the expressed genome of the AA1/5 isolate, which also caused disease on two infected cultivars of *B. juncea*. While we have not quantified disease symptoms on cultivars Sej‐2(2) and Pusa Jaikisan, it was observed that the latter cultivar was more susceptible. This observation is compatible with a recent report that classified Sej‐2(2) as moderately resistant and Pusa Jaikisan as moderately susceptible.^13^

### 4.2 Pathogen transcriptomics

This is the first study of both *A. brassicae* and *B. juncea* transcriptomics during fungal infection and host colonisation. Almost all annotated fungal genes (99.6%) were expressed during *in vitro* culture or *in planta* growth. In agreement with a previous study of *A. brassicae* transcriptomics,^16^ certain CAZymes and *NEP1* were highly expressed in colonised leaves, although the GO‐term carbohydrate metabolic process was enriched at 2 dpi and not at 4 dpi as previously described.^16^ Two expressed *NEP1* genes (A4220, A4322) were identified, but only A4220 was significantly higher expressed *in planta* than *in vitro*. A4322 corresponds to *AbrNLP2* that, unlike *AbrNLP1* (A4220), was reported to induce necrosis in host or non‐host leaves.^42^

Surprisingly, pathogen genes involved in endocytosis were down‐regulated in colonised leaf tissues. This could implicate a communication breakdown with the host or an interference with uptake of toxic molecules from the host, especially during later stages of infection. Although speculative, the possibility cannot be excluded that a reduction of endocytosis may interfere with delivery of extracellular vesicles that are known to be part of the penetration resistance pathway of host plants.^43^

### 4.3 Host transcriptomics

Expression of host genes encoding subunits of the two photosystems was suppressed after *A. brassicae* infection. As reported for *Alternaria solani* infection of potato cultivars,^44^ components of PS I (e.g., *PsaE*, *PsaF*, *PsaG*, *PsaH*, *PsaK*, *PsaN*), the oxygen‐evolving centre of PS II (*PsbO*, *PsbX*, *PsbY*) and PS II assembly (e.g., *Psb28*) were down‐regulated after *A. brassicae* challenge. Similarly to the reported study,^44^ differences between cultivars in down‐regulation of photosynthetic genes were observed here. The impact of necrotrophic pathogenesis on photosynthetic parameters using chlorophyll fluorometry has been documented to include decreases in effective quantum yield of PS II, non‐photochemical quenching and electron transport rate.^45^ Concomitant with a decrease in PS gene expression, OPPP genes were up‐regulated, presumably to maintain NADPH production for assimilation pathways.

Modifications of plant and pathogen cell walls play an important role during fungal penetration and subsequent colonisation of the host.^46^ Besides differential induction of host chitinases in the two *B. juncea* cultivars tested, a down‐regulation of callose biosynthetic genes was observed, especially in Sej‐2(2) during the late stage of infection. This is notable because callose deposition was reported to be associated with susceptibility to *A. brassicae*.^47^ Host‐derived pectate lyases can act as susceptibility factors to support fungal ingress.^48^ Down‐regulation of pectate lyase genes during *A. brassicae* colonisation, especially in Sej‐2(2), may therefore play a quantitative role in controlling disease progress.

Analysis of genes that were differently expressed in two cultivars during the earliest stage of infection identified separate sets of DEGs. Genes involved in jasmonic acid (JA) and auxin turnover were more strongly induced or expressed in Sej‐2(2) than in Pusa Jaikisan.^49^, ^50^ *JOX4* represents a jasmonate‐induced oxygenase that mediates down‐regulation of JA‐dependent responses.^49^ Similarly, *DAO2* contributes to attenuation of auxin signalling through a constitutive mechanism that is slower than amino acid conjugation.^50^ A gene involved in indole glucosinolate modification (*CYP81F3*) and implicated in defence against *Alternaria brassicicola* was also more strongly induced in Sej‐2(2) than in Pusa Jaikisan.^51^, ^52^ On the contrary, *PR‐3* like basic chitinase, *LIK1*, *CRK21* and *RLP35* were more strongly induced in Pusa Jaikisan than in Sej‐2(2). Induction of *PR‐3* chitinase by *A. brassicae* was reported in *A. thaliana*.^53^ *LIK1*, which is part of the chitin receptor complex,^54^ and *CRK21* were induced in a resistant *B. napus* line after inoculation with *Leptosphaeria maculans* (syn. *Plenodomus lingam*).^55^ Although the contribution of *RLP35* to immunity remains unknown, the expression of this gene is up‐regulated after treatment of *A. thaliana* with bacterial outer membrane vesicles.^56^ Suppression of *MLO2* in Pusa Jaikisan but not in Sej‐2(2) seems to be more consistent with down‐regulation of a susceptibility response.^57^ Collectively, these data suggest that different responses to *A. brassicae* inoculation are triggered in each of the cultivars, although their relative magnitudes and importance cannot readily be appreciated.

### 4.4 Validation using qPCR analysis of *PR‐4* an *PR‐5* expression

*PR‐4* and *PR‐5* were chosen for an independent validation of the quality of the RNA that was used for transcriptomics. Subtle differences between *PR‐4* and *PR‐5* expression after *A. brassicae* inoculation were observed in both *B. juncea* cultivars, although the overall trend of significantly induced expression of two copies of each of these genes was similar. No significant difference in cultivar‐dependent and pathogen‐induced expression was observed. These data are consistent with a role of these *PR* genes in defence as previously noted. *PR‐4* encodes a hevein‐like antimicrobial peptide (AMP) with six cysteine residues, belonging to the AMP2 category.^58^, ^59^ *PR‐5* encodes a secreted thaumatin superfamily protein with eight disulfide bonds that is involved in biotic and abiotic stress responses.^60^, ^61^

## 5 CONCLUSION

Here, the first *A. brassicae* genome of a European isolate is reported. In contrast to a previously published Indian isolate, the genome of the European isolate features far fewer genes (62% of the Indian complement). A possible reason for this is supernumerary chromosomes in the Indian isolate. Nonetheless, important pathogenicity functions are maintained and expressed in the European isolate. Aside from the perhaps expected induction of CAZymes and *NEP* genes, a novel down‐regulation of genes involved in endocytosis was observed, which could have important implications for host colonisation. Fungal attack down‐regulated photosystem genes with a complementary up‐regulation of OPPP genes in host cultivars. Host callose biosynthetic genes and pectate lyase genes were also suppressed, and this was discussed within the context of reduced susceptibility. A subset of DEGs differed between cultivars. Closer inspection identified distinct genes involved in defence pathways in the two cultivars, but without a clear signature that could explain small differences in genotypic disease susceptibility. These new findings advance our understanding of the *A. brassicae*–*B. juncea* pathosystem.

## CONFLICT OF INTEREST

The authors have no conflict of interest to declare.

## Supporting information


**Data S1.** Supporting Information.

## Data Availability

The DNA sequence data generated during this study are available from NCBI BioProject database accession PRJNA1313841, as SRA accessions SRR35269331 to SRR35269332, for BioSample accession SAMN50912061. The Whole Genome Shotgun project has been deposited at DDBJ/ENA/GenBank under the accession JBQWFL000000000. The version described in this paper is version JBQWFL010000000. The RNA‐sequencing data generated and analysed during this study are available from NCBI BioProject database accession PRJNA1311480, as SRA accessions SRR35172280 to SRR35172306, for BioSample accessions SAMN50808434 to SAMN50808458, SAMN50809920 to SAMN50809921.
