# Supplementary material for: First genome sequence of a European Alternaria brassicae isolate and genes involved in early development of alternaria leaf spot on Brassica juncea
Source: Pest Manag Sci. 2026 Jan 21;82(11):10841–54. doi: 10.1002/ps.70477 (PMC13665613; doi:10.1002/ps.70477)
Supplement: Supplementary file 1 [file PS-82-10841-s001.zip › Pest Management Science - PM-25-1750.R2.zip!/SupplementaryTables.zip!/SupplementaryTables/TableS1.docx]

**Supplementary Table x. Sequences used in phylogenetic analyses in the present study.**

|  |  |  | **Gene (GenBank accession numbers)** | | | | |
| --- | --- | --- | --- | --- | --- | --- | --- |
| **Species** | **Country** | **Source** | **ITS** | ***Alt a1*** | ***GADPH*** | ***pmATP*** |  |
| *Alt. alternata* | India | EGS 34-016 | AF347031 | KP275691 | AY278808 | JQ811979 |  |
| *Alt. arborescans* | USA | EGS 39-128 | AF347033 | AY563303 | AY278810 | JQ671880 |  |
| *Alt. tenuissima* | UK | EGS 34-015 | AF347032 | KP275690 | AY278809 | JQ811989 |  |
| *Alt. brassicae* | USA | EGS 38-032 | JQ693663 | AY563309 | AY562414 | JQ671847 |  |
| *Alt. brassicae* | India | J3 | CM016513.1:  29470-29999 | CM016505.1:  1086505-1086977 | CM016510.1:  837421-837991 | CM016510.1:  1261604-1262737 |  |
| *Alt. brassicae* | UK | AA1/5 | Contig 25:  373-902^a^ | Contig 9:  392139- 391667^a^ | Contig 6:  1564541- 1563971^a^ | Contig 6:  1140268- 1139136^a^ |  |
| *Alt. brassicola* | USA | EEB 2232 | AF229462 | AY563311 | AY278813 | JQ671843 |  |
| *Alt. japonica* | Serbia | Isolate 207 | MN173826 | MN175507 | MN175517 | MN175527 |  |
| *Stemphylium callistephi* | No data | EEB 1055 | AF229482 | AY563276 | AY278822 | JQ671769 |  |

^a^ NCBI BioProject database accession PRJNA1313841.
